# Supplementary material for: Interaction between ZMIZ2 and AR promotes prostate cancer proliferation in vitro and in vivo
Source: Cancer Biol Ther. 2025 Dec 23;27(1):2604936. doi: 10.1080/15384047.2025.2604936 (PMC12758332; doi:10.1080/15384047.2025.2604936)
Supplement: supplementary material — Graphical Abstract. [file KCBT_A_2604936_SM6363.docx]

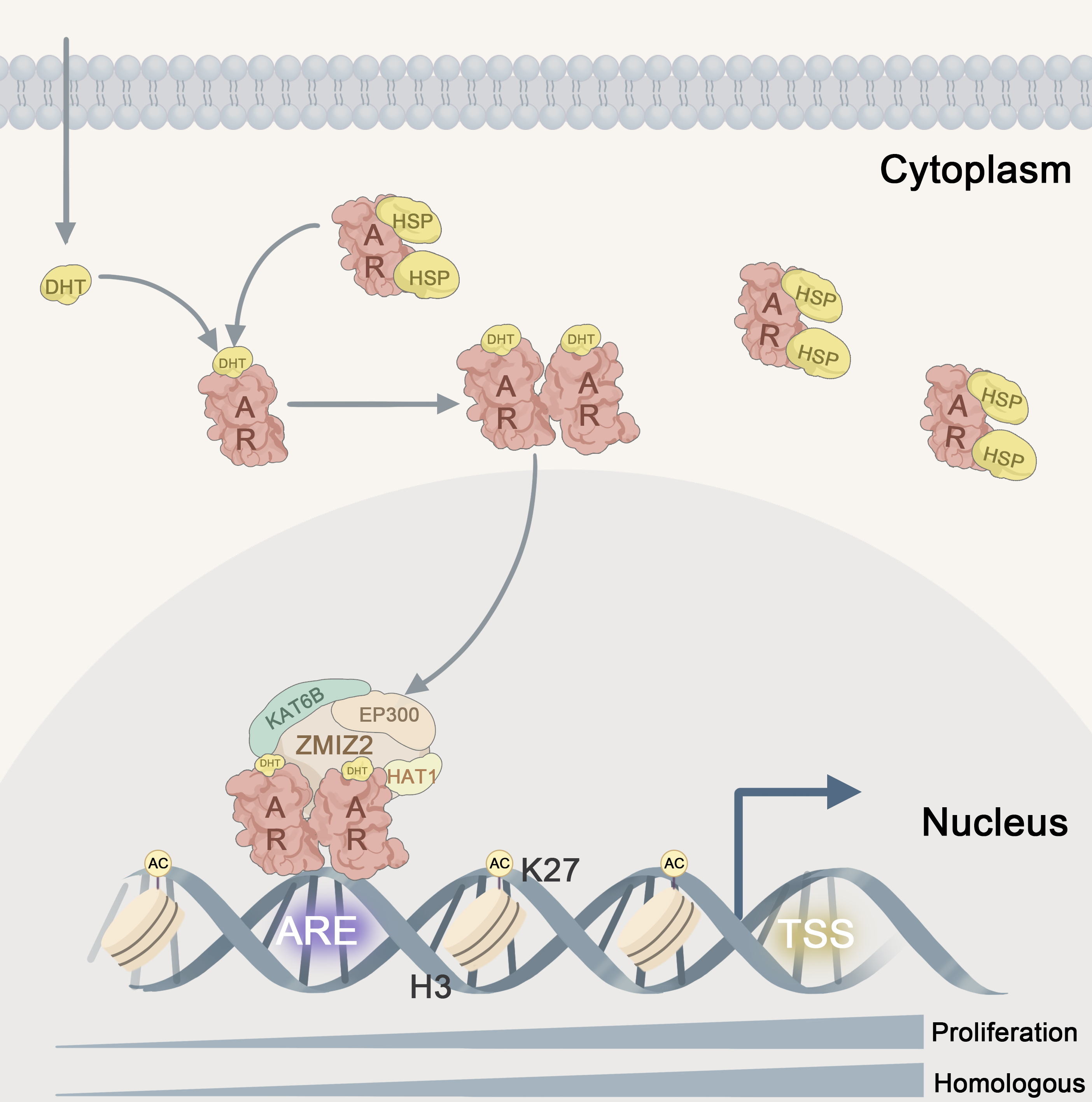


Graphical Abstract

This study focuses on prostate cancer, a malignancy of the prostate epithelium. As a transcriptional co-regulator of the androgen receptor (AR), ZMIZ2 plays an important role in the proliferation process of prostate cancer cells. Bioinformatics analysis reveals its elevated expression in prostate cancer tissues, positively correlated with the Gleason score. In vitro and in vivo experiments have demonstrated that ZMIZ2 promotes the proliferation of prostate cancer cells in an AR signaling - dependent manner. Mechanistically, as depicted in the figure, ZMIZ2 recruits acetylases (e.g., HAT1, KAT6B, EP300) and binds to AR's NTD. This complex binds to ARE near cell - cycle gene (CDK1, CCNA2, CCNE2) promoters, up - regulating transcription via the AR pathway and driving prostate cancer cell proliferation.
