# Supplementary material for: Interaction between ZMIZ2 and AR promotes prostate cancer proliferation in vitro and in vivo
Source: Cancer Biol Ther. 2025 Dec 23;27(1):2604936. doi: 10.1080/15384047.2025.2604936 (PMC12758332; doi:10.1080/15384047.2025.2604936)
Supplement: supplementary material — KCBT_S_2025_0764.R1_Source_Files. [file KCBT_A_2604936_SM6362.zip › 校稿可编辑图片/Figure 1/Figure Legend.docx]

**Figure 1.** ZMIZ2 is highly expressed in prostate cancer tissues, and its expression level is positively correlated with the Gleason score. (a) In the TCGA databases, the expression of ZMIZ2 in different types of tumors was compared with that in normal tissues. (b) ZMIZ2 expression in prostate adenocarcinoma (PRAD) tissues and normal tissues in the TCGA databases. (c) ZMIZ2 expression in PRAD tissues and their matched normal tissues in the TCGA databases. (d) ZMIZ2 expression in PRAD stratified by the patient's Gleason score. (e) Survival curves of patients with high and low ZMIZ2 expression in the TCGA databases. (f) ROC curves for distinguishing prostate cancer from normal prostate tissues in the TCGA database. (g) Tissue specimens of Benign Prostatic Hyperplasia (BPH) and Prostate Cancer (Pca) were collected and processed into pathological sections for subsequent microscopic analysis. HE staining was used to observe the structural changes in Pca tissue samples, and the expression level of ZMIZ2 in each group was examined by IHC staining. (h) Quantitative analysis of (g). (i) Immunohistochemical staining was performed to detect ZMIZ2 expression in tissue samples from each group. Distribution of patient samples by Gleason Grade Groups.Each group contained n=5 cases: Grade Group 1 (GS ≤6), Grade Group 2 (GS 3+4=7), Grade Group 3 (GS 4+3=7), Grade Group 4 (GS 8), and Grade Group 5 (GS 9-10). (j) Quantitative analysis of (j). Significant differences are indicated as: **p* < 0.05, ***p* < 0.01, and ****p* < 0.001; ns indicates not significant.
