# Supplementary material for: Interaction between ZMIZ2 and AR promotes prostate cancer proliferation in vitro and in vivo
Source: Cancer Biol Ther. 2025 Dec 23;27(1):2604936. doi: 10.1080/15384047.2025.2604936 (PMC12758332; doi:10.1080/15384047.2025.2604936)
Supplement: supplementary material — KCBT_S_2025_0764.R1_Source_Files. [file KCBT_A_2604936_SM6362.zip › 校稿可编辑图片/Figure 2/Figure Legend.docx]

**Figure 2.** ZMIZ2 promotes the proliferation of prostate cancer cells. (a) Western Blot analysis of ZMIZ2 protein expression levels in BPH1 and LNCaP cells. (b) qPCR analysis of ZMIZ2 mRNA levels in BPH1 and LNCaP cells. (c) Western Blot results showing the silencing efficiency of ZMIZ2 protein in cells transfected with ZMIZ2 interference plasmids. (d) qPCR results showing the silencing efficiency of ZMIZ2 protein in cells transfected with ZMIZ2 interference plasmids. (e) Western Blot analysis of ZMIZ2 protein expression levels after transfection with ZMIZ2 overexpression plasmids. (f) qPCR analysis of ZMIZ2 mRNA transcriptional levels after transfection with ZMIZ2 overexpression plasmids. (g - h) A clonogenic assay was employed to quantitatively evaluate the proliferative capacity of cells in different groups, visually presenting the cell growth dynamics and proliferation potential under various experimental conditions. (i) Cell viability was determined by the CCK - 8 assay. (j - k) An EdU assay was used to analyze cell proliferation. (l - m) Colony formation assays were performed to assess the proliferative capacities of cells in the respective groups. (n) Cell viability was determined by the CCK - 8 assay. (o - p) EdU assays were conducted to evaluate the proliferative levels of cells in each group. Significant differences are indicated as: **p* < 0.05, ***p* < 0.01, and ****p* < 0.001; ns indicates not significant; n = 3.
