# Supplementary material for: Interaction between ZMIZ2 and AR promotes prostate cancer proliferation in vitro and in vivo
Source: Cancer Biol Ther. 2025 Dec 23;27(1):2604936. doi: 10.1080/15384047.2025.2604936 (PMC12758332; doi:10.1080/15384047.2025.2604936)
Supplement: supplementary material — KCBT_S_2025_0764.R1_Source_Files. [file KCBT_A_2604936_SM6362.zip › 校稿可编辑图片/Figure 3/Figure Legend.docx]

**Figure 3.** ZMIZ2 promotes tumor cell proliferation through the Androgen Receptor (AR) signaling pathway. (a) A luciferase reporter gene assay was conducted to explore the regulatory role of ZMIZ2 in modulating the transcriptional activity of the PSA promoter in LNCaP cells. (b) A luciferase reporter gene assay was conducted to explore the regulatory role of ZMIZ2 in modulating the transcriptional activity of the PSA promoter in PC3 cells. (c) A luciferase reporter gene assay was used to detect the transcriptional activity of the PSA promoter when ZMIZ2 was overexpressed and AR was knocked down simultaneously. (d - e) Colony formation experiments were carried out to quantitatively assess the proliferative capacity of cells in each experimental group. Colonies were counted and statistically analyzed to evaluate differences in cell growth and clonogenic potential. (f) Cell viability was determined by the CCK - 8 assay. (g - h) An EdU assay was employed to analyze cell proliferation levels in each group by visualizing and quantifying EdU - incorporated cells. Significant differences are indicated as: **p* < 0.05, ***p* < 0.01, and ****p* < 0.001; ns indicates not significant; n = 3.
