# Supplementary material for: Interaction between ZMIZ2 and AR promotes prostate cancer proliferation in vitro and in vivo
Source: Cancer Biol Ther. 2025 Dec 23;27(1):2604936. doi: 10.1080/15384047.2025.2604936 (PMC12758332; doi:10.1080/15384047.2025.2604936)
Supplement: supplementary material — KCBT_S_2025_0764.R1_Source_Files. [file KCBT_A_2604936_SM6362.zip › 校稿可编辑图片/Figure 4/Figure Legend.docx]

**Figure 4.** ZMIZ2 functions as a transcriptional co - regulator of AR, specifically binding to the N - terminal domain (NTD) of AR. (a) Protein docking analysis between ZMIZ2 and AR. (b) Immunofluorescence experiments were conducted to observe the expression locations of ZMIZ2 and AR in LNCaP cells upon stimulation with DHT. (c - d) Co - IP experiments were performed to detect the binding interaction between ZMIZ2 and AR in LNCaP and 22RV1. (e) The PDBePISA website was utilized to predict the binding energies of different truncated forms of ZMIZ2 with respect to AR. (f) The pymol software was employed to construct the protein docking model of ZMIZ2 (residues 392 - 527) and AR. (g) The PDBePISA website was used to predict the binding energies of different truncated forms of AR with full - length ZMIZ2. (h) Protein docking analysis between AR (residues 1 - 333) and ZMIZ2. (i) GST pull - down experiments were carried out to detect the binding of different truncated forms of ZMIZ2 to AR. (j) GST pull - down experiments were carried out to detect the binding of different truncated forms of AR to ZMIZ2. Significant differences are indicated as: **p* < 0.05, ***p* < 0.01, and ****p* < 0.001; ns indicates not significant.
