# Supplementary material for: Interaction between ZMIZ2 and AR promotes prostate cancer proliferation in vitro and in vivo
Source: Cancer Biol Ther. 2025 Dec 23;27(1):2604936. doi: 10.1080/15384047.2025.2604936 (PMC12758332; doi:10.1080/15384047.2025.2604936)
Supplement: supplementary material — KCBT_S_2025_0764.R1_Source_Files. [file KCBT_A_2604936_SM6362.zip › 校稿可编辑图片/Figure 5/Figure Legend.docx]

**Figure 5.** To uncover the molecular regulatory network underlying the functions of ZMIZ2 and AR, RNA - seq analysis was meticulously performed to identify the common downstream target genes of these two factors. (a) RNA - seq analysis of differentially expressed genes after ZMIZ2 silencing or AR silencing.
(b) Venn diagram analysis of genes commonly upregulated by ZMIZ2 and AR.
(c) KEGG analysis of genes commonly upregulated by ZMIZ2 and AR. (d) GO analysis of genes commonly upregulated by ZMIZ2 and AR. (e) Heatmap of the expression levels of cell cycle - related genes after ZMIZ2 silencing or AR silencing. (f - g) A flow cytometry assay was employed to determine the cell cycle distribution. (h) QPCR was utilized to assess the mRNA transcriptional levels of cell cycle - related genes. (i) Western blot analysis was carried out to detect the protein expression levels of CDK1, CCNA2, and CCNE2 in each sample group. Significant differences are indicated as: **p* < 0.05, ***p* < 0.01, and ****p* < 0.001; ns indicates not significant; n = 3.
