# Supplementary material for: Interaction between ZMIZ2 and AR promotes prostate cancer proliferation in vitro and in vivo
Source: Cancer Biol Ther. 2025 Dec 23;27(1):2604936. doi: 10.1080/15384047.2025.2604936 (PMC12758332; doi:10.1080/15384047.2025.2604936)
Supplement: supplementary material — KCBT_S_2025_0764.R1_Source_Files. [file KCBT_A_2604936_SM6362.zip › 校稿可编辑图片/Figure 6/Figure Legend.docx]

**Figure 6.** ZMIZ2 recruits acetyltransferases to bind to AR, forming a transcriptional complex. (a) Flow chart of IP - MS. (b) GO analysis of proteins interacting with ZMIZ2. (c) Protein sequences of EP300, KAT6B, and HAT1 detected by the IP - MS experiment. (d) Protein docking analysis of ZMIZ2, AR, and EP300. (e) Protein docking analysis of ZMIZ2, AR, and KAT6B. (f) Protein docking analysis of ZMIZ2, AR, and HAT1. (g) The PDBePISA website was used to calculate the binding energies among ZMIZ2, AR, and EP300. (h) The PDBePISA website was used to calculate the binding energies among ZMIZ2, AR, and KAT6B. (i) The PDBePISA website was used to calculate the binding energies among ZMIZ2, AR, and HAT1. (j) IP experiments were performed to detect the binding interaction between ZMIZ2 and acetylases in the presence or absence of DHT. (k) IP experiments were performed to detect the binding interaction between AR and acetylases in the presence or absence of DHT.
(l - m) IP experiments were performed to detect the binding interaction between AR and acetylases after ZMIZ2 silencing. (n) Western Blot was used to detect the levels of H3K27ac, H2AK5ac, and H4K5ac after knocking down ZMIZ2. Significant differences are indicated as: **p* < 0.05, ***p* < 0.01, and ****p* < 0.001; ns indicates not significant.
