# Supplementary material for: Interaction between ZMIZ2 and AR promotes prostate cancer proliferation in vitro and in vivo
Source: Cancer Biol Ther. 2025 Dec 23;27(1):2604936. doi: 10.1080/15384047.2025.2604936 (PMC12758332; doi:10.1080/15384047.2025.2604936)
Supplement: supplementary material — KCBT_S_2025_0764.R1_Source_Files. [file KCBT_A_2604936_SM6362.zip › 校稿可编辑图片/Figure 7/Figure Legend.docx]

**Figure 7.** Depletion of ZMIZ2 expression is associated with reduced AR enrichment on the promoters of downstream target genes, accompanied by a concurrent decrease in H3K27ac levels. (a) Flow chart of the ChIP experiment. (b) The binding sites of AR on the promoters of CDK1, CCNA2, and CCNE2. (c - h) ChIP analysis of AR enrichment on the CDK1, CCNA2, and CCNE2 promoters and H3K27ac levels. Significant differences are indicated as: **p* < 0.05, ***p* < 0.01, and ****p* < 0.001; ns indicates not significant; n = 3.
