# Supplementary material for: Interaction between ZMIZ2 and AR promotes prostate cancer proliferation in vitro and in vivo
Source: Cancer Biol Ther. 2025 Dec 23;27(1):2604936. doi: 10.1080/15384047.2025.2604936 (PMC12758332; doi:10.1080/15384047.2025.2604936)
Supplement: supplementary material — KCBT_S_2025_0764.R1_Source_Files. [file KCBT_A_2604936_SM6362.zip › 校稿可编辑图片/Figure Legend/Figure 8图注.docx]

**Figure 8.** Orthotopic prostate inoculation of RM-1 cells transfected with ZMIZ2-OE or ZMIZ2-OE+AR-shRNA in wild-type mice to assess tumor proliferation. (a) RM-1 cells were inoculated into the prostate of mice; tumors were excised surgically after 14 days under pentobarbital sodium anesthesia. (b) Representative images of tumors from each group. (c) Quantitative analysis of tumor weights. (d) Measurement of tumor volumes. (e) Immunohistochemical (IHC) staining of tumor sections for ZMIZ2, AR, and cell cycle-related proteins. (f - g) Densitometric quantification of IHC results. Significance is denoted as: **p* < 0.05, ***p* < 0.01, ****p* < 0.001; ns, not significant; n = 6 per group.
