# Supplementary material for: Interaction between ZMIZ2 and AR promotes prostate cancer proliferation in vitro and in vivo
Source: Cancer Biol Ther. 2025 Dec 23;27(1):2604936. doi: 10.1080/15384047.2025.2604936 (PMC12758332; doi:10.1080/15384047.2025.2604936)
Supplement: supplementary material — KCBT_S_2025_0764.R1_Source_Files. [file KCBT_A_2604936_SM6362.zip › 校稿可编辑图片/main document.docx]

**Interaction between ZMIZ2 and AR Promotes Prostate Cancer Proliferation in Vitro and in Vivo**

Jing-ze Yu^a,†^, Xiao-pan Zou^b,†^, Xiao-bei Wu^c^, Yang-yang Cui^a^, Tan Wei^d^, Jun Di^e^, Xue-chao Feng^a,*^, Xiao-meng Li^a,d,*^

^a^ School of Life Sciences, Northeast Normal University, Changchun, Jilin 130024, China

^b^ Jilin Provincial People's Hospital, Changchun, Jilin 130000, China

^c^ Shenzhen University General Hospital，Shenzhen, Guangdong 518055, China

^d^ KingMed Laboratory College, Guangzhou Medical University, Guangzhou, Guangdong 511400, China

^e^ Beijing university of Chinese medicine hospital in Shenzhen,Shenzhen, Guangdong 518100, China

* Corresponding author.

*E-mail addresses:* fengxc997@nenu.edu.cn (X. Feng), lixm441@nenu.edu.cn (X. Li)

^†^ These authors have contributed equally to this work and share the first authorship.

**ABSTRACT**

**Background：**ZMIZ2, an androgen receptor (AR) transcriptional co-regulator, promotes prostate cancer cell proliferation by interacting with AR to upregulate genes associated with cell proliferation; however, its specific cooperative mechanisms remain unclear. This study aims to elucidate these mechanisms.

**Materials and Methods：**We analyzed the expression level and prognostic significance of ZMIZ2 in prostate cancer using bioinformatics methods. ZMIZ2 expression and its correlation with the Gleason score were analyzed using clinical samples. LNCaP cells with ZMIZ2 overexpression or AR knockdown were employed to evaluate cell proliferation. RNA-seq, qPCR, Western blot, and co-immunoprecipitation were used to explore the molecular mechanisms. In vivo xenograft models were utilized to validate the effects.

**Results：**ZMIZ2 expression was significantly higher in prostate cancer tissues and positively correlated with the Gleason score. Overexpressing ZMIZ2 robustly promoted LNCaP cell growth, but this promoting effect was dramatically lessened in the absence of AR expression. Mechanistically, ZMIZ2 recruited multiple acetyltransferases and formed a transcriptional complex with the N-terminal domain of AR, which bound to the promoters of cell cycle-related genes CDK1, CCNA2, and CCNE2, leading to upregulated transcription. Both in vitro cell culture experiments and in vivo models supported ZMIZ2’s role in promoting proliferation.

**Conclusion：**ZMIZ2 promotes prostate cancer cell proliferation through the AR signaling pathway by regulating key cell cycle genes, highlighting it as a potential therapeutic target.

**Keywords***:* Prostate cancer; Androgen receptor（AR）; ZMIZ2; Acetyltransferase; Cell cycle

**Introduction**

Prostate cancer (PCa) is a malignant tumor arising from the prostate epithelium. Prostate cancer primarily affects middle-aged and elderly men, and its incidence increases steadily with age[1]. Patients with prostate cancer often experience symptoms such as dysuria, frequent urination, painful urination, and hematuria, which seriously affect their quality of life[2]. Moreover, prostate cancer cells can easily spread to other tissues and organs through the blood and lymphatic systems, causing various complications.The pathogenic factors of prostate cancer have not been fully clarified. Currently, most researchers believe that the development of prostate cancer is associated with factors such as age, environment, dietary habits, heredity, and abnormal secretion of sex hormones, among others[3].

A large number of studies have shown that the abnormal activation of androgen receptor (AR) signaling is a key factor in the occurrence and development of prostate cancer[4-6]. Androgen deprivation therapy or androgen antagonists such as bicalutamide and enzalutamide can effectively inhibit the progression of prostate cancer in clinical practice[7, 8]. AR is a member of steroid hormone receptor family, and contains four functional domains: the ligand binding domain (LBD), DNA binding domain (DBD), hinge domain (HR) and N-terminal domain (NTD). The binding of 5 α-dihydrotestosterone (DHT) to the LBD of AR induces a conformational change, leading to the dissociation of chaperone proteins, facilitating the interaction between N-terminal and C-terminal of AR and dimerization, after which the complex translocates to the nucleus. Once inside the nucleus, the AR dimer binds to the androgen response elements (AREs) in the promoter regions of the AR target genes[9, 10].The NTD and LBD domains of AR recruit numerous transcriptional co-regulators and chromatin-modifying proteins, forming transcriptional complexes that interact with the promoter regions of AR target genes, thereby up-regulating their transcription of these genes. Over-activation of AR signaling can increase the transcription of many oncogenes promoting the proliferation, invasion and metastasis of prostate cancer cells[11, 12].

In recent years, increasing attention has been given to the identification and characterization of transcription co-regulatory factors of AR[13]. ZMIZ2 is a transcriptional co-regulator of AR that contains a highly homologous MIZ domain to the PIAS family. However, studies on the function of ZMIZ2 remain limited; its expression pattern, clinical significance and regulatory role in tumor-associated signaling pathways have not been fully elucidated. ZMIZ2 is highly expressed in the prostate, heart, brain, pancreas, and ovaries, with lower expression in other tissues. It is also upregulated in various cancer types, and studies have shown that the high ZMIZ2 expression is positively correlated with tumor cell proliferation[14-16]. Yang et al. reported that silencing ZMIZ2 significantly inhibited the proliferation, cell cycle process, migration and invasion of HCC cells in vitro[17]. Similarly, Zou et al. found that elevated ZMIZ2 expression of ZMIZ2 promoted the proliferation of breast cancer cells[18].

In previous studies, we found that ZMIZ2 interacts with AR to enhance AR-mediated transcription, and interacts with the SWI/SNF-like BAF complex to facilitate chromatin remodeling[19]. Huang et al. reported that AR and ZMIZ2 were co-localized in CV-1 cells and LNCaP cells, and that ZMIZ2 overexpression enhanced the transcriptional activity of PSA[20]. Collectively, these findings indicate that ZMIZ2 is closely associated with the occurrence and progression of prostate cancer and is likely a key protein contributing to the excessive proliferation of prostate cancer cells.

The molecular mechanism by which ZMIZ2 promotes prostate cancer development has therefore drawn our attention. In this study, we found that ZMIZ2 was highly expressed in prostate cancer tissues, and that its overexpression promoted prostate cancer cell proliferation. This proliferation-promoting effect was dependent on AR signaling. Moreover, we found that ZMIZ2 can recruit several acetyltransferases, including EP300, KAT6B and HAT1, to regulate H3K27 acetylation. ZMIZ2 interacts with AR in prostate cancer cells to promote the transcription of cell proliferation-related genes, thereby enhancing the proliferation of prostate cancer cells.

**Materials and methods**

***Clinical samples***

Patient samples, benign prostatic hyperplasia tissues (n=5) and prostate cancer tissues (n=25), were collected from Jilin Province People's Hospital. The collaboration with the hospital was conducted under the ethical approval (Approval No. 202502021) from the Ethics Committee of Northeast Normal University. The hospital ensured that all patients provided written informed consent, and sample collection strictly adhered to ethical guidelines (including the Declaration of Helsinki) as reviewed and approved by the ethics committee.

***Cell and cell culture***

LNCaP cells were cultured in Roswell Park Memorial Institute 1640 (RPMI-1640; Sigma) supplemented with 10% foetal bovine serum (FBS, Thermo Fisher) and 1% penicillin/streptomycin (Invitrogen). LNCaP were transfected with ZMIZ2 over-expressed plasmid (A gift from professor Zijie Sun, Stanford University, United States of America) or AR-shRNA plasmid using Lipofecatmine (Thermo Fishe) as a manufacturer's instruction.

***Colony formation assay***

We inoculated different groups of cells in the six-well plate, with about 400 cells per hole. Cultivate with 1640 media containing 10% FBS for two weeks. When there are visible colonies formed, discarded the medium, using PBS to wash the cells twice. After being fixed with 4% paraformaldehyde for 20 min, cells were washed with PBS and stained with Giemsa stain for 30 min. Finally, cells were washed with PBS twice and the positive colonies that contained > 50 cells were counted under ordinary optical microscope.

***CCK-8 assay***

In brief, 5000 cells were inoculated into 96-well microplates and cultured overnight. Cell viability was detected by transfection of different plasmids at 12h, 24h, 36h and 48h. Add 10ul CCK-8 to each well, and then incubate at 37°C for 2-4 h. Finally, the absorbance was detected at 450 nm wavelength by enzyme-labeled instrument. The cell viability was calculated according to the absorbance ratio between the experimental group and the control group.

***EdU cell proliferation assay***

We inoculated the cells of each group into 6-well plates, and stained them with EdU after 0.48 h.. Add EdU working solution (20 μM) to 1 mL of culture solution to make the final concentration of EdU in culture solution 10 μM m.. The cells were incubated in a 37ºC cell incubator for 2 hours. Next, the culture medium was removed, and 1 ml of 4% paraformaldehyde fixed solution was added and fixed at room temperature for 15 min. Remove the stationary solution, and wash the cells with 1 mL PBS for 3-5 min each time. Remove the washing liquid, and incubate with 1 mL PBS containing 0. 3% TritonX-100 for 10-15 min at room temperature. Discard the liquid, and wash the cells with 1 mL PBS for 1-2 times, 3-5 min each time. Remove PBS, add 0. 20 ml of Click reaction solution to each well, and gently shake the culture plate to ensure that the reaction mixture can cover the sample evenly. Incubate at room temperature in the dark for 30 min. Discard the Click reaction solution and wash it with PBS for 3-5 min each time. Add 1 μL DAPI(1000×) into 1 mL PBS, shake well, add 300 μL to each hole, incubate in the dark for 5-10 min, stain the nucleus, discard DAPI dye solution, and wash with PBS for 3-5 min each time. Finally, observe and take photos under the fluorescent microscope.

***Flow cytometry for cell cycle***

LNCaP cells were fixed with 70% ethanol at 4 °C for 2 h, and subsequently reacted in staining buffer containing PI (7Sea Biotech, China) and RNase at 37 °C for 30 min. Cell cycle was finally tested by a Flow Cytometer (Beckman, USA).LNCaP cells were fixed in 70% ethanol at 4℃ for 2 hours, then placed in dyeing buffer containing PI and RNase at 37℃ for 30 minutes, and finally the cell cycle was detected by flow cytometry.

***RNA extraction and quantitative real-time PCR***

Total RNA was isolated from cells in line with the manufacturer's protocols using RNAiso (Takara, Tokyo, Japan). The purity and concentration of the total RNA were gauged by a spectrophotometer (Nanodrop 2000, Thermo Fisher Scientific, Waltham, MA, USA) at wavelengths of 260 and 280 nm. For all samples, the absorption ratios (260/280 nm) fell within the range of 1.80 - 2.00. Subsequently, cDNA was generated with a Primer ScriptTM RT Reagent Kit containing gDNA Eraser (Takara, Tokyo, Japan). Transcript levels were quantified via qPCR, employing the Quant Studio 3 (Thermo Fisher Scientific, Waltham, MA, USA). The PCR procedure was as follows: an initial denaturation at 95°C for 5 min, followed by 40 cycles of 95°C for 5 s and 60°C for 34 s. Relative gene expression was computed by applying the 2−ΔΔCt (threshold cycle difference) method, with GAPDH serving as the housekeeping gene. All measurements were carried out at least 3 times.

The primers were as follows: forward primer 5'-GTCTCCTCTGACTTCAACAGCG-3’and reverse primer 5'-ACCACCCTGTTGCTGTAGCCAA-3’for GAPDH; forward primer 5'-CCTGGCTGTAAGCAACCATGTC-3' and reverse primer 5'-GCCAGTTGGTGTTCATCTGCCG-3' for ZMIZ2; forward primer 5'-ATGGTGAGCAGAGTGCCCTATC-3' and reverse primer 5'-ATGGTCCCTGGCAGTCTCCAAA-3' for AR; forward primer 5'-GGAAACCAGGAAGCCTAGCATC-3' and reverse primer 5'-GGATGATTCAGTGCCATTTTGCC-3' for CDK1; forward primer 5'-CTCTACACAGTCACGGGACAAAG-3' and reverse primer 5'-CTGTGGTGCTTTGAGGTAGGTC' for CCNA2; forward primer 5'-CTTACGTCACTGATGGTGCTTGC-3' and reverse

primer 5'-CTTGGAGAAAGAGATTTAGCCAGG' for CCNE2.

***Immunohistochemical (IHC) staining***

Human prostate cancer tissue slices were dewaxed in xylene and gradually rehydrated. After antigen repair, peroxidase was blocked with hydrogen peroxide (3%). Then, it was incubated with 10% goat serum for 30 minutes at room temperature. The ZMIZ2 antibody (Novus Biologicals, LLC, Centennial, CO, USA), AR antibody (Santa Cruz Biotechnology, Dallas, TX, USA), CDK1 antibody (Santa Cruz Biotechnology, Dallas, TX, USA), CCNA2 antibody (Santa Cruz Biotechnology, Dallas, TX, USA), CCNE2 antibody (Santa Cruz Biotechnology, Dallas, TX, USA) were added and incubated overnight at 4℃, and then incubated with HRP conjugated goat anti-rabbit IgG (Thermo Fisher Scientific, Waltham, MA, USA) at 37℃ for 30 minutes. Immune complexes were observed by using 3,3-diaminophenylhydrazine. The glass slide was counterstained with light hematoxylin, dehydrated and covered with glass. Following microscopic observation and image acquisition, the IHC staining intensity was objectively quantified using Image J software through measurement of the mean optical density specifically within positively stained regions in each field of view.

***Immunofluorescence (IF) staining***

The cultured cells underwent three washes with phosphate - buffered saline (PBS). Subsequently, they were fixed at room temperature for 20 minutes in 4% paraformaldehyde (PFA, with a pH of 7.4). After permeabilization with 0.2% Triton X-100, the cells were blocked with 5% bovine serum albumin (BSA) for 1 hour. Next, the primary antibodies (anti - ZMIZ2 and anti - AR) were diluted at a ratio of 1:150 and incubated with the cells overnight at 4°C. After being washed three times with PBS, the cells were incubated with fluorescein isothiocyanate (FITC)-conjugated goat anti - mouse IgG (diluted 1:100) for 2 hours at room temperature. Post - washing, the cells were stained with 4',6-diamidino-2-phenylindole (DAPI) for 3 minutes. Finally, an inverted fluorescence microscope was utilized to observe the cells.

***Western blot***

Total protein was extracted by RIPA. BCA kit was used to determine the protein concentration of each group. Then add SDS buffer and cook 5min boiling water for 5 minutes. Finally, SDS-PAGE electrophoresis was performed. After electrophoresis, protein was transferred to PVDF and sealed with 5% milk for 1h. Add following primary antibodies and incubate overnight：ZMIZ2 (Novus Biologicals, LLC, Centennial, CO, USA), AR (Santa Cruz Biotechnology, Dallas, TX, USA), CDK1 (Santa Cruz Biotechnology, Dallas, TX, USA), CCNA2 (Santa Cruz Biotechnology, Dallas, TX, USA), CCNE2 (Santa Cruz Biotechnology, Dallas, TX, USA). The protein blots were visualized by employing either a goat anti - rabbit IgG HRP - conjugated secondary antibody or a goat anti - mouse IgG HRP - conjugated secondary antibody (purchased from CST, located in Danvers, Massachusetts, USA). The detection process was carried out using a FluorChem M Fluorescent Imaging System (specifically the Tanon 5200 model from Tanon Science & Technology Co., Ltd., based in Shanghai, China), along with the Tanon™ High - sig ECL Western Blotting Substrate from the same company (Tanon Science & Technology Co., Ltd., Shanghai, China).

***Co-Immunoprecipitation assay***

Inoculate LNCaP cells into a 10 - cm cell culture dish. When the cell growth density reaches 80%, remove the culture medium and gently rinse the cells twice with PBS. Add cell lysate and then collect the supernatant. Add the AR antibody to the supernatant and incubate it at 4 °C for 8 hours. Then, add 30 μL of protein A/G to the lysate and incubate it at 4 °C for 4 hours. Centrifuge the solution, remove the supernatant, and add lysis buffer to wash the agarose beads 5 times. Next, add SDS sample buffer to the agarose beads and boil them in water at 100 °C for 5 minutes. Finally, perform SDS - PAGE electrophoresis.

***In vivo tumor formation assays***

This study employed two distinct mouse tumor models to investigate tumor formation in vivo. Male BALB/c nude mice (6 weeks old, 18-20 g) were utilized for establishing a subcutaneous xenograft model using LNCaP cells (n=5), while male C57BL/6 mice (6 weeks old, 20-22 g) served for developing an orthotopic model using RM-1 cells (n=6). The animals were housed under specific pathogen-free conditions and randomly assigned using a random number table method to three experimental groups: Control group (inoculated with control plasmid-transfected cells), ZMIZ2-oe group (inoculated with ZMIZ2-overexpressing cells), and ZMIZ2-oe+AR-shRNA group (inoculated with cells co-expressing ZMIZ2-oe and AR-shRNA). All animal experiments were approved by the Ethics Committee of Northeast Normal University (Approval No. 202502021) and conducted in accordance with institutional guidelines.

Stable cell lines were established through transfection followed by G418 selection. For the orthotopic prostate cancer model, C57BL/6 mice were anesthetized by intraperitoneal injection of sodium pentobarbital (50 mg/kg) and received an injection of RM-1 cells (5×10⁴ cells in 10 μL PBS) directly into the prostate lobe. For the subcutaneous xenograft model, nude mice were anesthetized with sodium pentobarbital (50 mg/kg, intraperitoneal) and subcutaneously injected with LNCaP cells (1×10⁶ cells in 100 μL PBS) into the right upper limb. All surgical procedures were performed under aseptic conditions with proper wound closure.

Mice were euthanized two weeks (orthotopic model) or three weeks (subcutaneous model) post-injection via an intraperitoneal overdose of sodium pentobarbital (150 mg/kg). Tumor volume and tumor mass were measured as primary outcome measures. Pathological specimens were collected for immunohistochemical analysis.

***Statistical analysis***

All statistical analyses were conducted using GraphPad Prism 9. To compare data between two groups, the unpaired two - tailed t - test was employed. Across all analyses, the thresholds for statistical significance were defined as follows: **p* < 0.05, ***p* < 0.01, and ****p* < 0.001. All data are presented in the form of the mean ± the standard error of the mean (SEM). Each mean value was derived from a minimum of three independent experiments.

**Results**

***Elevated expression of ZMIZ2 in prostate tumors***

At present, more and more research results show that ZMIZ2 is highly expressed in a variety of tumor tissues[20, 21]. Pan-cancer analysis revealed that the expression of ZMIZ2 was highly expressed in many types of cancers, such as bladder urothelial carcinoma, breast cancer, rectum adenocarcinoma, stomach adenocarcinoma, and head and neck tumor (Figure 1a). We compared the mRNA levels of ZMIZ2 between 499 prostate cancer tissues and 52 normal prostate tissues, which were retrieved from TCGA. The results demonstrated that the expression level of ZMIZ2 was significantly elevated in the 499 prostate cancer tissues (*p* < 0.001) (Figure 1b). Moreover, the data from 52 paired samples indicated that the mRNA expression level of ZMIZ2 in cancer tissues was remarkably higher than that in adjacent non-cancerous tissues (*p* < 0.001) (Figure 1c). The expression level of ZMIZ2 was positively correlated with the Gleason score (Figure 1d). Meanwhile, the increase of ZMIZ2 expression indicated the poor survival of patients (Figure 1e). Furthermore, the ROC curve indicated that ZMIZ2 expression had predictive power with an area under the curve (AUC) of 0.775 (95% confidence interval [CI] = 0.718–0.832) to discriminate prostate cancer tissues from normal tissues (Figure 1f).

To further verify the high expression of ZMIZ2 in prostate cancer, tissue samples were collected from patients with prostatic hyperplasia and prostate cancer and made into pathological sections. HE staining revealed that, in contrast to benign prostatic hyperplasia, prostate cancer tissue exhibited an obvious accumulation of cancer cells, a decreased number of acini, and an obvious shrinkage of acini. Immunohistochemical experiments were also conducted using the ZMIZ2 antibody. The results demonstrated that the expression level of ZMIZ2 in prostate cancer tissue was significantly increased, and the positive staining area essentially coincided with the accumulation area of cancer cells (Figure 1g). To investigate the correlation between ZMIZ2 expression levels and prostate cancer malignancy, a series of prostate cancer specimens with different Gleason Grade Groups were collected and subjected to immunohistochemical staining. The results indicated that ZMIZ2 was highly expressed in cancer tissues but relatively low in adjacent non-cancerous tissues. Notably, as the Gleason Grade Group advanced, the expression level of ZMIZ2 significantly increased (*p* < 0.05) (Figure 1i).

***ZMIZ2 promotes the proliferation of prostate cancer cells***

The expression levels of ZMIZ2 in benign prostatic hyperplasia cells (BPH1) and prostate cancer cells (LNCaP) were detected. The results demonstrated a significantly elevated expression level of ZMIZ2 in LNCaP cells (*p* < 0.001) (Figure 2a,b). Subsequently, ZMIZ2-knocked down and ZMIZ2-overexpressed LNCaP cell lines were constructed. The efficiency of gene editing was verified by Western blot and q-PCR experiments, confirming the successful establishment of the cell lines with ZMIZ2 silenced and overexpressed (Figure 2c - f).

Subsequently, a colony formation assay was performed to analyze the influence of ZMIZ2 on the proliferation of prostate cancer cells. The results revealed that silencing ZMIZ2 could suppress the clone numbers of LNCaP cells to different extents, both in the presence and absence of dihydrotestosterone (DHT) stimulation. Notably, under DHT stimulation, the inhibitory effect of silencing ZMIZ2 on the clone numbers of LNCaP cells was more pronounced (*p* < 0.001) (Figure 2g, h). The CCK-8 assay results indicated that silencing ZMIZ2 could remarkably inhibit the proliferation rate of LNCaP cells under DHT stimulation (Figure 2i). The EdU staining experiment yielded results consistent with the aforementioned ones, demonstrating that silencing ZMIZ2 could inhibit the proliferation of LNCaP cells, and this inhibitory effect was more prominent under DHT stimulation (Figure 2j,k).

Subsequently, we overexpressed ZMIZ2 in PC3 cells, an androgen receptor (AR)-negative prostate cancer cell line, and detected proliferation-related indicators. We found that PC3 cells were insensitive to DHT stimulation, and overexpression of ZMIZ2 failed to upregulate the proliferation capacity of PC3 cells（Figure 2l-p）. These results suggest that the promotion of prostate cancer cell proliferation by ZMIZ2 depends on the AR signaling pathway.

***ZMIZ2 promotes the proliferation of prostate cancer cells through the AR signaling pathway***

To elucidate the potential role of ZMIZ2 in promoting the proliferation of prostate cancer cells via the AR signaling pathway, we initially probed into the regulatory effect of ZMIZ2 on the transcription of prostate-specific antigen (PSA), a well-recognized downstream target gene of AR[21, 22]. The results of the luciferase reporter assay showed that ZMIZ2 significantly enhanced the transcriptional activity of the PSA promoter in LNCaP cells (Figure 3a, c), whereas it had no such effect in PC3 cells (Figure 3b). ZMIZ2-OE plasmid and AR-shRNA plasmid were simultaneously transfected into LNCaP cells. The results of the clone formation assay indicated that the proliferation ability of LNCaP cells was remarkably enhanced following transfection with the ZMIZ2-OE plasmid. However, when both the ZMIZ2-OE plasmid and AR-shRNA plasmid were transfected, the proliferation ability of LNCaP cells was evidently inhibited (Figure 3d,e). The CCK-8 assay results demonstrated that high expression of ZMIZ2 augmented the viability of LNCaP cells. Nevertheless, when both plasmids were transfected, the capacity of ZMIZ2 to promote the viability of LNCaP cells was substantially attenuated (Figure 3f). The EdU cell proliferation experiment also revealed that when AR was inhibited, the ability of ZMIZ2 to up-regulate DNA replication in LNCaP cells was significantly impaired (Figure 3g,h).

***Interaction between ZMIZ2 and AR***

In this study, we obtained the protein structure files of ZMIZ2 and AR from the UniProt database. Then, the binding energy of ZMIZ2 and AR was predicted via the PDBePISA website. Using the PyMOL software, we constructed the docking structural model of ZMIZ2 and AR and precisely marked the binding sites (Figure 4a).

The results demonstrated a relatively strong binding interaction between ZMIZ2 and AR, with a binding energy of -53.60 Kcal/mol. Under normal circumstances, ZMIZ2 is expressed within the cell nucleus, while AR is mostly expressed in the cytoplasm. However, upon stimulation by DHT, AR is induced to translocate into the nucleus and bind to ZMIZ2. Immunofluorescence results indicated that, under the stimulation of DHT (1×10⁻⁸ M), AR and ZMIZ2 exhibited co-localization within the cell nucleus (Figure 4b). A direct physical interaction was validated by co-immunoprecipitation (Co-IP) in LNCaP (Figure 4c). To further generalize our findings, we performed Co-IP in 22RV1 cells, another model of prostate cancer, and similarly confirmed the endogenous interaction between ZMIZ2 and AR (Figure 4d). To probe the peptide segments of ZMIZ2-AR binding, we constructed truncated structural models of ZMIZ2 and AR and predicted their binding energies. The results demonstrated that ZMIZ2 (392-527) exhibited a relatively high binding energy with the full-length AR, with the binding energy being -41.90 Kcal/mol (Figure 4e, f). Meanwhile, AR (1-333) also showed a relatively high binding energy with the full-length ZMIZ2, and the binding energy was -46.20 Kcal/mol (Figure 4g, h). To further dissect the binding mechanism and corroborate these findings, plasmids harboring GST tags were engineered for diverse truncated peptide segments of both ZMIZ2 and AR. Subsequent Pull-down assays unequivocally demonstrated that the fragments ZMIZ2 (392-527) and AR (1-333) play pivotal roles in mediating the binding interaction between ZMIZ2 and AR (Figure 4i, j).

***ZMIZ2 and AR coordinately regulate the transcription of cell cycle genes***

To further identify the common target genes of ZMIZ2 and AR, we established an LNCaP cell line with deficient ZMIZ2 expression was deficient and an LNCaP cell line in which AR expression was lacking. The alternation of transcriptomes in LNCaP cells due to the deficiency of ZMIZ2 expression or the deficiency of AR expression was monitored by RNA-sequencing analysis. After ZMIZ2 silencing, 128 genes exhibit up - regulated transcription, while 249 genes show down - regulated transcription. Upon AR silencing, the number of genes with up - regulated transcription is 192, and that with down - regulated transcription is 277 (Figure 5a). We then determined the intersection of genes with down - regulated transcription following ZMIZ2 silencing and those following AR silencing (Figure 5b). It was found that there are 63 genes co - regulated by ZMIZ2 and AR. KEGG analysis of these 63 genes revealed that most of them are enriched in pathways such as Cell cycle, Homologous recombination, Prostate cancer, Breast cancer, and Ras signaling pathway (Figure 5c). The GO analysis indicated that the majority of these co - regulated genes participate in biological processes including cell cycle, DNA recombination, and DNA damage checkpoint (Figure 5d). These findings imply that ZMIZ2 may cooperate with AR to promote the transcription of cell - cycle genes. The transcription levels of cell-cycle-related genes co-regulated by ZMIZ2 and AR are presented as a heatmap (Figure 5e). As expected, the transcription levels of most cell - cycle - related genes decreased significantly after silencing of either ZMIZ2 or AR.

To further explore whether the regulation of LNCaP cell - cycle progression by ZMIZ2 is AR - dependent, we examined the cell - cycle progression of LNCaP cells in different groups using flow cytometry. The results demonstrated that when ZMIZ2 was over - expressed, the number of cells in the S - phase increased. However, when AR was simultaneously silenced with ZMIZ2 over - expression, the effect of ZMIZ2 on increasing the number of S - phase cells was significantly attenuated(Figure 5f,g).

Subsequently, we extracted total RNA from each group of cells and measured the mRNA levels of cell-cycle genes by q-PCR. The results indicated that the transcription levels of all cell-cycle-related genes were up-regulated to varying extents when ZMIZ2 was over-expressed. However, there was no significant difference in the transcription levels of cell - cycle - related genes between the group with ZMIZ2 over - expression and AR knockdown and the control group(Figure 5h). Among these genes, CDK1, CCNA2, and CCNE2 showed the most prominent transcriptional changes. We therefore assessed the corresponding protein levels of CDK1, CCNA2, and CCNE2 by Western blot. The results indicated that the overexpression of ZMIZ2 led to the upregulation of the expression levels of CDK1, CCNA2, and CCNE2, while the knockdown of AR nullified this effect(Figure 5i). These results suggest that ZMIZ2 upregulates the transcription of the cell cycle-related genes CDK1, CCNA2 and CCNE2 through AR.

***ZMIZ2 recruits acetylase to bind with AR***

To further investigate the potential interaction protein network of ZMIZ2 and the molecular mechanism underlying its regulation of AR signaling, we conducted IP-MS experiments(Figure 6a) and performed GO analysis on the proteins that ZMIZ2 binds to. The results show that most of the proteins bound by ZMIZ2 are involved in regulating life - related processes such as DNA repair, cell cycle, and androgen metabolic process. In addition, the proteins bound by ZMIZ2 are also involved in the formation of histone acetyltransferase complexes (Figure 6b). We found that ZMIZ2 can bind to various acetylases, including EP300, KAT6B, and HAT1 (Figure 6c). We constructed a binding model of ZMIZ2, AR, and various acetylases using the pymol software, and the results show that ZMIZ2 can recruit EP300, KAT6B, and HAT1 to bind to the AF1 of AR (Figure 6d - f).Next, we calculated the binding energies between EP300, KAT6B, HAT1 and ZMIZ2, AR using the PDBePISA database. The results showed that the binding energies between AR and acetyltransferases were higher in the presence of ZMIZ2(Figure 6g-i). Co - IP analysis has confirmed our speculation that ZMIZ2 can bind to EP300, KAT6B, and HAT1 with or without DHT stimulation (Figure 6j). Under DHT stimulation, AR can bind more EP300 and HAT1 (Figure 6k). However, when ZMIZ2 is knocked out, the binding of AR to acetylases is significantly weakened (Figure 6m). Histone acetyltransferases such as EP300, KAT6B, and HAT1 can regulate the levels of H3k27ac, H2Ak5ac, and H4k5ac. To explore the relationship between ZMIZ2 and histone acetylation, we measured the levels of H3k27ac, H2Ak5ac, and H4k5ac, and the results show that the silencing of ZMIZ2 significantly inhibits the level of H3k27ac (Figure 6n).

***ZMIZ2 upregulates the binding effect of AR to the promoters of downstream target genes***

In previous experiments, we have established that ZMIZ2 up-regulates the mRNA levels of CDK1, CCNA2, and CCNE2 in an AR - dependent manner. Subsequently, to elucidate the molecular mechanism underlying the regulation of CDK1, CCNA2, and CCNE2 expression by the ZMIZ2 - AR axis, we confirmed our hypothesis through ChIP experiments. Initially, we retrieved the conserved sequences of androgen response elements (AREs) from the JASPAR database (https://jaspar.genereg.net/). Concurrently, we explored the chromatin immunoprecipitation sequencing (ChIP-seq) data of AR within the TCGA database. The results showed that the binding sites of AR on the promoters of CDK1, CCNA2, and CCNE2 highly overlap with the H3K27ac sites (Figure S1). Subsequently, in combination with the PROMO database (https://alggen.lsi.upc.es/CGI-bin/PROMO_v3/PROMO/PROMO_init.cgi?DirDB=TF_8.3), we predicted the binding sites of AR on the promoters of CDK1, CCNA2, and CCNE2. Thereafter, primers were designed corresponding to the distinct AREs(Figure 7b). The influence of ZMIZ2 on the binding ability between AR and AREs was verified via ChIP experiments. The results of the ChIP assays demonstrated that following the silencing of ZMIZ2, the enrichment of AR on the AREs of the CDK1 promoter was markedly diminished. Simultaneously, the level of H3K27ac histone acetylation on the CDK1 promoter was also significantly rEdUced. Analogously, on the CCNA2 promoter, the AREs exhibited a decreased AR enrichment upon ZMIZ2 depletion, accompanied by a decline in the H3K27ac level, with the most prominent effect observed at ARE2 of the CCNA2 promoter. Consistent results were obtained on the CCNE2 promoter, where the silencing of ZMIZ2 led to a substantial rEdUction in AR enrichment and a concomitant decrease in the H3K27ac modification level(Figure 7c-h). Collectively, these results suggest that ZMIZ2 and AR engage in a transcriptional synergy. Specifically, ZMIZ2, acting as a transcriptional co - regulator of AR, is capable of recruiting acetylase to interact with AR, thereby jointly forming a transcriptional complex. This complex upregulates the acetylation level of the promoters of downstream target genes, augments the binding affinity between AR and AREs, and consequently initiates the transcription of CDK1, CCNA2, and CCNE2.

***ZMIZ2 overexpression promotes prostate cancer progression in vivo***

While previous experiments have confirmed that ZMIZ2 promotes prostate cancer cell proliferation via the AR signaling pathway, discrepancies exist between in vitro culture conditions (with artificial DHT intervention) and in vivo physiological states. Subcutaneous xenograft tumor assays using LNCaP cells in nude mice further validated that ZMIZ2 drives prostate tumor proliferation through AR signaling (Figure S2). However, since subcutaneous xenografts cannot fully recapitulate the natural progression of prostate cancer, we employed an orthotopic prostate tumor model using RM-1 cells to utilize the mice's endogenous androgen secretion system, thereby dynamically mimicking AR signaling activation patterns in clinical patients. RM-1 cells with ZMIZ2-OE or ZMIZ2-OE combined with AR knockdown (ZMIZ2-OE+AR-shRNA) were inoculated into the prostate of wild-type mice. Fourteen days after inoculation, mice were anesthetized with pentobarbital sodium, and tumor tissues were surgically excised (Figure 8a). Comprehensive analyses including photographic documentation, volumetric measurement, and weight quantification revealed that ZMIZ2-OE tumors exhibited accelerated in vivo proliferation, whereas AR knockdown in ZMIZ2-OE tumors significantly attenuated ZMIZ2-mediated pro-proliferative effects (Figure 8b-d). Immunohistochemical staining of pathological sections showed that CDK1, CCNA2, and CCNE2 protein levels were upregulated in a ZMIZ2-dependent manner in ZMIZ2-OE tumors, while ZMIZ2-OE+AR-shRNA tumors displayed no significant differences in these markers compared to controls (Figure 8e).

**Discussion**

Zinc finger MIZ domain-containing protein 2 (ZMIZ2), which is a PIAS-like protein, has a MIZ domain highly homologous to that of the PIAS protein family and is predominantly localized in the nuclei of prostate epithelial cells, where it interacts with the AR. Studies have demonstrated that in the nucleus, ZMIZ2 can form a complex with AR and co-localize within replication foci. Additionally, ZMIZ2 is able to enhance the transcriptional activity of AR other nuclear hormone receptors[23].

Our research findings demonstrate that ZMIZ2 plays a significant role in the occurrence and development of prostate cancer. The expression level of ZMIZ2 is positively correlated with the malignancy grade of prostate cancer. High ZMIZ2 expression promotes the proliferation of prostate cancer cells.However, its role in promoting tumor cell proliferation is dependent on AR signaling. The absence of AR expression remarkably attenuates the effect of ZMIZ2 in promoting tumor cell proliferation.

In the absence of DHT stimulation, the AR is mostly expressed in the cytoplasm and binds to heat shock family proteins to prevent the AR from being degraded via the proteasome pathway[24-26]. However, under the condition of DHT stimulation, the ligand-binding domain (LBD) of AR will bind to DHT[27, 28], thereby changing the spatial conformation of the AR protein. This causes the AR to dissociate from the heat shock protein complex and enter the nucleus. Then, it forms dimers and binds to the androgen response elements (AREs) in the promoter regions of target genes[29, 30]. Subsequently, co-regulatory factors are recruited to form a transcriptional complex, which can then activate or inhibit the transcription of target genes[31, 32].

In molecular biology, the ZMIZ2 protein has been demonstrated to exhibit a predominant nuclear localization pattern. After AR translocation into the nucleus following activation, ZMIZ2 directly interacts with the activation function 1 (AF1) domain of AR. This binding event serves as a crucial regulatory node, modulating the transcriptional activation potency of AR towards its downstream target genes, which play pivotal roles in various physiological and pathological processes relevant to androgen-responsive tissues.Our experimental findings demonstrate that ZMIZ2 is capable of recruiting multiple acetylases, including EP300, KAT6B1, and HAT1, to form a transcriptional complex together with AR. Specifically, EP300 and KAT6B1 can recognize and bind to the chromatin regions that contain histone H3. Utilizing acetyl-CoA as the acetyl group donor, they add an acetyl group to the lysine 27 residue of histone H3, thereby increasing the level of H3K27ac[33, 34]. This acetylation modifies the chromatin structure, making it more accessible and creating favorable conditions for transcription factors and RNA polymerase to bind to DNA, thus facilitating gene transcription[34-36].

Our experimental findings validate this hypothesis. ZMIZ2 is expressed in the nuclei of prostate cancer cells and binds to acetylases such as EP300. When AR translocates into the nucleus, ZMIZ2, along with acetylases, binds to the AF1 domain of AR, forming a transcriptional complex. Following the binding of AR's DBD region to downstream target gene promoters, acetylases can enhance the level of H3k27ac, subsequently promoting the transcriptional upregulation of cell cycle-related genes, including CDK1, CCNA2, and CCNE2.

In summary, our research has comprehensively elucidates the multifaceted role of ZMIZ2 in prostate cancer. As a PIAS - like protein, ZMIZ2 interacts with AR in the nucleus of prostate epithelial cells, enhancing AR's transcriptional activity. Its high expression positively correlates with prostate cancer malignancy and promotes cancer cell proliferation in an AR-dependent manner. Mechanistically, ZMIZ2 recruits acetylases to form a transcriptional complex with AR, modifying histone H3 at lysine 27 through acetylation, which in turn upregulates cell cycle-related genes. These findings not only validate our initial hypothesis but also provide novel insights into the molecular mechanisms underlying prostate cancer development, potentially opening new avenues for targeted therapies against this disease.

**Disclosure statement**

The authors declare that they have no known competing financial interests or personal relationships that could have appeared to influenced the work reported in this paper.

**Funding**

Our research was supported by the National Natural Science Foundation of China (Grant Number 31870758), the Natural Science Foundation of Jilin Province (20200201127JC) and Jilin Provincial Science and Technology Department Project (202401007JJ).

**Authors’ contributions**

Xiao-meng Li, Jing-ze Yu and Jun Di designed the entire project. Jing-ze Yu，Xiao-pan Zou, Xue-chao Feng and Yang-yang Cui participated in and completed all the experiments and wrote the paper. Xiao-bei Wu, Tan Wei and Xiao-pan Zou were responsible for animal feeding and data analysis. Jing-ze Yu, Xiao-bei Wu and Xiao-pan Zou have made equal contributions to this article. The authors have read and approved the final version of the manuscript.

**Consent for publication**

All authors approved the final manuscript and the submission to this journal.

**Data availability statement**

The raw data of the results showed in this study can be obtained from the corresponding author according to reasonable requirements.

**Ethics approval and consent to participate**

This study was approved by the Ethics Committee of Northeast Normal University (Approval No. 202502021). The approval covered the entire research process, including sample collection at Jilin Province People's Hospital. All patients recruited at the hospital provided written informed consent, after being fully informed of the study details. Experiments were conducted in accordance with relevant ethical guidelines, including the Declaration of Helsinki.

**References**

1 Robinson MJ, Ding Z, Pitt C et al. The Amount of BCL6 in B Cells Shortly after Antigen Engagement Determines Their Representation in Subsequent Germinal Centers. Cell Reports 2020; 30 (5): 1530-1541.e1534.

2 Gauthe M, Sargos P, Benziane ON et al. Restaging of Patients with Persistently Elevated Prostate-specific Antigen After Radical Prostatectomy Using [68Ga]-PSMA-11 Positron Emission Tomography/Computed Tomography: Impact on Disease Management. European urology 2022 (4): 81.

3 Mashele S. Prostate Cancer Review: Genetics, Diagnosis, Treatment Options, and Alternative Approaches. Molecules 2022; 27.

4 Kostos L, Tran B, Azad AA. Combination of PARP Inhibitors and Androgen Receptor Pathway Inhibitors in Metastatic Castration-Resistant Prostate Cancer. Drugs 2024; 84 (9): 1093-1109.

5 Kim J, Park S, Kim S et al. Enhancing the anticancer effect of androgen deprivation therapy by monocarboxylate transporter 1 inhibitor in prostate cancer cells. Prostate 2024; 84 (9).

6 Shengqiang, Yu, Shujie et al. Androgen receptor in human prostate cancer-associated fibroblasts promotes prostate cancer epithelial cell growth and invasion. Medical Oncology 2013.

7 Feng DC, Zhu WZ, Wang J et al. The implications of single-cell RNA-seq analysis in prostate cancer: unraveling tumor heterogeneity, therapeutic implications and pathways towards personalized therapy. Military Medical Research 2024; 11.

8 Paris PL, Weinberg V, Simko J et al. Preliminary evaluation of prostate cancer metastatic risk biomarkers. Int J Biol Markers 2005; 20 (3): 141-145.

9 Mitsiades, N. A Road Map to Comprehensive Androgen Receptor Axis Targeting for Castration-Resistant Prostate Cancer. Cancer Research 2013; 73 (15): 4599-4605.

10 Tabibian M, Moghaddam FS, Motevaseli E et al. Targeting mRNA-coding genes in prostate cancer using CRISPR/Cas9 technology with a special focus on androgen receptor signaling. Cell Communication & Signaling 2024; 22 (1).

11 Lonergan PE, Tindall DJ. Androgen receptor signaling in prostate cancer development and progression. Journal of Carcinogenesis 2011; 10 (1): 20.

12 Martin SK, Banuelos CA, Sadar MD et al. N-terminal targeting of androgen receptor variant enhances response of castration resistant prostate cancer to taxane chemotherapy. Molecular Oncology 2015.

13 Meyer S, Wang YH, Pérez-Escrivà P et al. Backbone 1H, 15N, 13C NMR assignment of the 518–627 fragment of the androgen receptor encompassing N-terminal and DNA binding domains. Biomolecular Nmr Assignments 2016; 10: 175-178.

14 Peng Y, Lee J, Zhu C et al. A Novel Role for Protein Inhibitor of Activated STAT (PIAS) Proteins in Modulating the Activity of Zimp7, a Novel PIAS-like Protein, in Androgen Receptor-mediated Transcription. Journal of Biological Chemistry 2010; 285 (15): 11465-11475.

15 Mohr SE, Boswell RE. Zimp encodes a homologue of mouse Miz1 and PIAS3 and is an essential gene in Drosophila melanogaster. Gene 1999; 229 (1-2): 109-116.

16 Ding Y, Ning Y, Kang H et al. ZMIZ2 facilitates hepatocellular carcinoma progression via LEF1 mediated activation of Wnt/β-catenin pathway. Experimental Hematology & Oncology 2024; 13 (1).

17 Lee, S. H, Zhu et al. Identification of a novel role of ZMIZ2 protein in regulating the activity of the Wnt/β-catenin signaling pathway. The Journal of biological chemistry 2013.

18 Zou X, Liu Y, Di J et al. ZMIZ2 promotes the development of triple-receptor negative breast cancer. Cancer cell international 2022; 22 (1): 52.

19 Chun-Yin H, Jason B, Xiaoyu L et al. hZimp7, a novel PIAS-like protein, enhances androgen receptor-mediated transcription and interacts with SWI/SNF-like BAF complexes. Molecular Endocrinology 2005; 19 (12): 2915-2929.

20 Moreno-Ayala R, Schnabel D, Salas-Vidal E et al. PIAS-like protein Zimp7 is required for the restriction of the zebrafish organizer and mesoderm development. Developmental Biology 2015; 403 (1): 89-100.

21 Liu M, Pan H, Zhang F et al. Identification of TNM stage-specific genes in lung adenocarcinoma by genome-wide expression profiling. Oncology letters 2013; 6 (3): 763-768.

22 Zhu Y, Gu L, Lin X et al. LINC00265 promotes colorectal tumorigenesis via ZMIZ2 and USP7-mediated stabilization of β-catenin. Cell Death & Differentiation.

23 Rodriguez-Magadán H, Merino E, Schnabel D et al. Spatial and temporal expression of Zimp7 and Zimp10 PIAS-like proteins in the developing mouse embryo. Gene Expression Patterns 2008; 8 (3): 206-213.

24 Jianzhuo L, Xueqi F, Subing C et al. Membrane-associated androgen receptor (AR) potentiates its transcriptional activities by activating heat shock protein 27 (HSP27). Journal of Biological Chemistry 2018; 293: jbc.RA118.003075.

25 Liu C, Lou W, Yang JC et al. Proteostasis by STUB1/HSP70 complex controls sensitivity to androgen receptor targeted therapy in advanced prostate cancer. Nature Communications 2018; 9 (1).

26 Centenera MM, Carter SL, Gillis JL et al. Co-targeting AR and HSP90 suppresses prostate cancer cell growth and prevents resistance mechanisms. Endocrine Related Cancer 2015; 22 (5): 805-818.

27 Chen, Jinbo, Chou et al. Androgen dihydrotestosterone (DHT) promotes the bladder cancer nuclear AR-negative cell invasion via a newly identified membrane androgen receptor (mAR-SLC39A9)-mediated G alpha i protein/MAPK/MMP9 intracellular signaling.

28 Jones JO, An WF, Diamond MI. AR inhibitors identified by high-throughput microscopy detection of conformational change and subcellular localization. Acs Chemical Biology 2009; 4 (3): 199-208.

29 Zhang H, Li X, Yang Y et al. Significance and Mechanism of Androgen Receptor (AR) Overexpression and AR-mTOR Crosstalk in Hepatocellular Carcinoma. Hepatology 2017.

30 Chen L, Zhang Z, Han Q, Barun K.Rodrigues, LeticiaZboril, EmilyAdhikari, RashmiKo, Su-HyukLi, XinYoshida, Shawn R.Xue, PengyaSmith, EmilieXu, KexinWang, QianbenHuang, Tim Hui-MingChong, ShashaLiu, Zhijie. Hormone-induced enhancer assembly requires an optimal level of hormone receptor multivalent interactions. Molecular cell 2023; 83 (19): 3438-3456.

31 Cutress ML, Whitaker HC, Mills IG et al. Structural basis for the nuclear import of the human androgen receptor. Journal of Cell Science 2008; 121 (7): 957-968.

32 Purayil HT, Zhang Y, Black JB et al. Nuclear βArrestin1 regulates androgen receptor function in castration resistant prostate cancer. Oncogene.

33 Hannon E, Marzi SJ, Schalkwyk LS et al. Genetic risk variants for brain disorders are enriched in cortical H3K27ac domains. Molecular Brain 2019; 12 (1).

34 Wu J, Gong L, Li Y et al. SGK1 aggravates idiopathic pulmonary fibrosis by triggering H3k27ac-mediated macrophage reprogramming and disturbing immune homeostasis. International Journal of Biological Sciences 2024; 20 (3).

35 Kang TG, Lan X, Zebley CC et al. Epigenetic regulators of clonal hematopoiesis control CD8 T cell stemness during immunotherapy.

36 Krug B, Jay ND, Harutyunyan AS et al. Pervasive H3K27 Acetylation Leads to ERV Expression and a Therapeutic Vulnerability in H3K27M Gliomas. Cancer Cell 2019; 35 (5): 782-797.e788.

**Figure 1.** ZMIZ2 is highly expressed in prostate cancer tissues, and its expression level is positively correlated with the Gleason score. (a) In the TCGA databases, the expression of ZMIZ2 in different types of tumors was compared with that in normal tissues. (b) ZMIZ2 expression in prostate adenocarcinoma (PRAD) tissues and normal tissues in the TCGA databases. (c) ZMIZ2 expression in PRAD tissues and their matched normal tissues in the TCGA databases. (d) ZMIZ2 expression in PRAD stratified by the patient's Gleason score. (e) Survival curves of patients with high and low ZMIZ2 expression in the TCGA databases. (f) ROC curves for distinguishing prostate cancer from normal prostate tissues in the TCGA database. (g) Tissue specimens of Benign Prostatic Hyperplasia (BPH) and Prostate Cancer (Pca) were collected and processed into pathological sections for subsequent microscopic analysis. HE staining was used to observe the structural changes in Pca tissue samples, and the expression level of ZMIZ2 in each group was examined by IHC staining. (h) Quantitative analysis of (g). (i) Immunohistochemical staining was performed to detect ZMIZ2 expression in tissue samples from each group. Distribution of patient samples by Gleason Grade Groups.Each group contained n=5 cases: Grade Group 1 (GS ≤6), Grade Group 2 (GS 3+4=7), Grade Group 3 (GS 4+3=7), Grade Group 4 (GS 8), and Grade Group 5 (GS 9-10). (j) Quantitative analysis of (j). Significant differences are indicated as: **p* < 0.05, ***p* < 0.01, and ****p* < 0.001; ns indicates not significant.

**Figure 2.** ZMIZ2 promotes the proliferation of prostate cancer cells. (a) Western Blot analysis of ZMIZ2 protein expression levels in BPH1 and LNCaP cells. (b) qPCR analysis of ZMIZ2 mRNA levels in BPH1 and LNCaP cells. (c) Western Blot results showing the silencing efficiency of ZMIZ2 protein in cells transfected with ZMIZ2 interference plasmids. (d) qPCR results showing the silencing efficiency of ZMIZ2 protein in cells transfected with ZMIZ2 interference plasmids. (e) Western Blot analysis of ZMIZ2 protein expression levels after transfection with ZMIZ2 overexpression plasmids. (f) qPCR analysis of ZMIZ2 mRNA transcriptional levels after transfection with ZMIZ2 overexpression plasmids. (g - h) A clonogenic assay was employed to quantitatively evaluate the proliferative capacity of cells in different groups, visually presenting the cell growth dynamics and proliferation potential under various experimental conditions. (i) Cell viability was determined by the CCK - 8 assay. (j - k) An EdU assay was used to analyze cell proliferation. (l - m) Colony formation assays were performed to assess the proliferative capacities of cells in the respective groups. (n) Cell viability was determined by the CCK - 8 assay. (o - p) EdU assays were conducted to evaluate the proliferative levels of cells in each group. Significant differences are indicated as: **p* < 0.05, ***p* < 0.01, and ****p* < 0.001; ns indicates not significant; n = 3.

**Figure 3.** ZMIZ2 promotes tumor cell proliferation through the Androgen Receptor (AR) signaling pathway. (a) A luciferase reporter gene assay was conducted to explore the regulatory role of ZMIZ2 in modulating the transcriptional activity of the PSA promoter in LNCaP cells. (b) A luciferase reporter gene assay was conducted to explore the regulatory role of ZMIZ2 in modulating the transcriptional activity of the PSA promoter in PC3 cells. (c) A luciferase reporter gene assay was used to detect the transcriptional activity of the PSA promoter when ZMIZ2 was overexpressed and AR was knocked down simultaneously. (d - e) Colony formation experiments were carried out to quantitatively assess the proliferative capacity of cells in each experimental group. Colonies were counted and statistically analyzed to evaluate differences in cell growth and clonogenic potential. (f) Cell viability was determined by the CCK - 8 assay. (g - h) An EdU assay was employed to analyze cell proliferation levels in each group by visualizing and quantifying EdU - incorporated cells. Significant differences are indicated as: **p* < 0.05, ***p* < 0.01, and ****p* < 0.001; ns indicates not significant; n = 3.

**Figure 4.** ZMIZ2 functions as a transcriptional co - regulator of AR, specifically binding to the N - terminal domain (NTD) of AR. (a) Protein docking analysis between ZMIZ2 and AR. (b) Immunofluorescence experiments were conducted to observe the expression locations of ZMIZ2 and AR in LNCaP cells upon stimulation with DHT. (c - d) Co - IP experiments were performed to detect the binding interaction between ZMIZ2 and AR in LNCaP and 22RV1. (e) The PDBePISA website was utilized to predict the binding energies of different truncated forms of ZMIZ2 with respect to AR. (f) The pymol software was employed to construct the protein docking model of ZMIZ2 (residues 392 - 527) and AR. (g) The PDBePISA website was used to predict the binding energies of different truncated forms of AR with full - length ZMIZ2. (h) Protein docking analysis between AR (residues 1 - 333) and ZMIZ2. (i) GST pull - down experiments were carried out to detect the binding of different truncated forms of ZMIZ2 to AR. (j) GST pull - down experiments were carried out to detect the binding of different truncated forms of AR to ZMIZ2. Significant differences are indicated as: **p* < 0.05, ***p* < 0.01, and ****p* < 0.001; ns indicates not significant.

**Figure 5.** To uncover the molecular regulatory network underlying the functions of ZMIZ2 and AR, RNA - seq analysis was meticulously performed to identify the common downstream target genes of these two factors. (a) RNA - seq analysis of differentially expressed genes after ZMIZ2 silencing or AR silencing.
(b) Venn diagram analysis of genes commonly upregulated by ZMIZ2 and AR.
(c) KEGG analysis of genes commonly upregulated by ZMIZ2 and AR. (d) GO analysis of genes commonly upregulated by ZMIZ2 and AR. (e) Heatmap of the expression levels of cell cycle - related genes after ZMIZ2 silencing or AR silencing. (f - g) A flow cytometry assay was employed to determine the cell cycle distribution. (h) QPCR was utilized to assess the mRNA transcriptional levels of cell cycle - related genes. (i) Western blot analysis was carried out to detect the protein expression levels of CDK1, CCNA2, and CCNE2 in each sample group. Significant differences are indicated as: **p* < 0.05, ***p* < 0.01, and ****p* < 0.001; ns indicates not significant; n = 3.

**Figure 6.** ZMIZ2 recruits acetyltransferases to bind to AR, forming a transcriptional complex. (a) Flow chart of IP - MS. (b) GO analysis of proteins interacting with ZMIZ2. (c) Protein sequences of EP300, KAT6B, and HAT1 detected by the IP - MS experiment. (d) Protein docking analysis of ZMIZ2, AR, and EP300. (e) Protein docking analysis of ZMIZ2, AR, and KAT6B. (f) Protein docking analysis of ZMIZ2, AR, and HAT1. (g) The PDBePISA website was used to calculate the binding energies among ZMIZ2, AR, and EP300. (h) The PDBePISA website was used to calculate the binding energies among ZMIZ2, AR, and KAT6B. (i) The PDBePISA website was used to calculate the binding energies among ZMIZ2, AR, and HAT1. (j) IP experiments were performed to detect the binding interaction between ZMIZ2 and acetylases in the presence or absence of DHT. (k) IP experiments were performed to detect the binding interaction between AR and acetylases in the presence or absence of DHT.
(l - m) IP experiments were performed to detect the binding interaction between AR and acetylases after ZMIZ2 silencing. (n) Western Blot was used to detect the levels of H3K27ac, H2AK5ac, and H4K5ac after knocking down ZMIZ2. Significant differences are indicated as: **p* < 0.05, ***p* < 0.01, and ****p* < 0.001; ns indicates not significant.

**Figure 7.** Depletion of ZMIZ2 expression is associated with reduced AR enrichment on the promoters of downstream target genes, accompanied by a concurrent decrease in H3K27ac levels. (a) Flow chart of the ChIP experiment. (b) The binding sites of AR on the promoters of CDK1, CCNA2, and CCNE2. (c - h) ChIP analysis of AR enrichment on the CDK1, CCNA2, and CCNE2 promoters and H3K27ac levels. Significant differences are indicated as: **p* < 0.05, ***p* < 0.01, and ****p* < 0.001; ns indicates not significant; n = 3.

**Figure 8.** Orthotopic prostate inoculation of RM-1 cells transfected with ZMIZ2-OE or ZMIZ2-OE+AR-shRNA in wild-type mice to assess tumor proliferation. (a) RM-1 cells were inoculated into the prostate of mice; tumors were excised surgically after 14 days under pentobarbital sodium anesthesia. (b) Representative images of tumors from each group. (c) Quantitative analysis of tumor weights. (d) Measurement of tumor volumes. (e) Immunohistochemical (IHC) staining of tumor sections for ZMIZ2, AR, and cell cycle-related proteins. (f - g) Densitometric quantification of IHC results. Significance is denoted as: **p* < 0.05, ***p* < 0.01, ****p* < 0.001; ns, not significant; n = 6 per group.
