# Supplementary material for: Interaction between ZMIZ2 and AR promotes prostate cancer proliferation in vitro and in vivo
Source: Cancer Biol Ther. 2025 Dec 23;27(1):2604936. doi: 10.1080/15384047.2025.2604936 (PMC12758332; doi:10.1080/15384047.2025.2604936)
Supplement: supplementary material — KCBT_S_2025_0764.R1_Source_Files. [file KCBT_A_2604936_SM6362.zip › 校稿可编辑图片/Supplementary Data/Supplementary Data.docx]

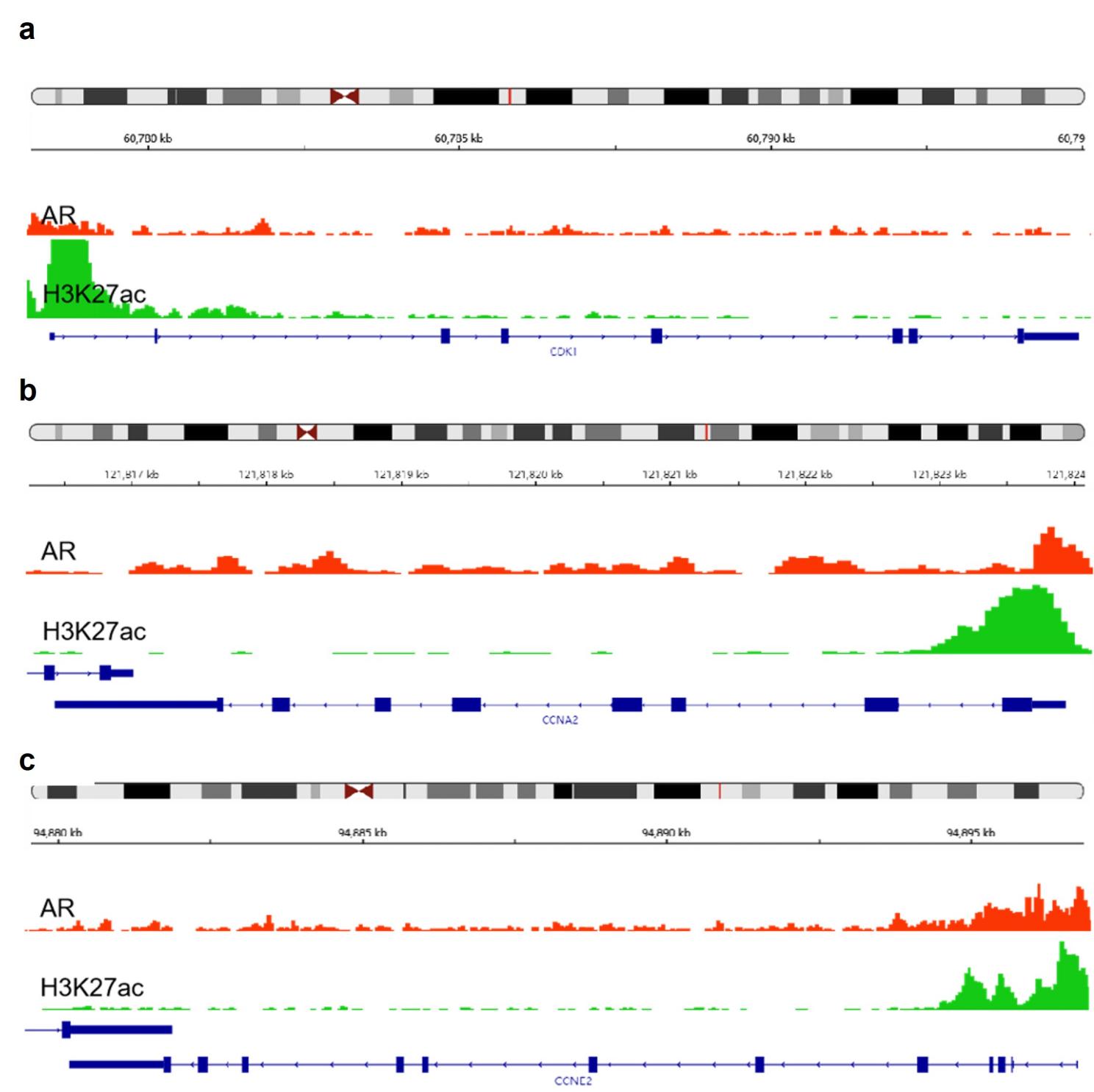


**Figure S1.** The binding positions of AR and H3K27ac on the promoters of downstream target genes highly overlap. (a - c) The binding sites of AR and the H3K27ac sites on the promoters of CDK1, CCNA2, and CCNE2.


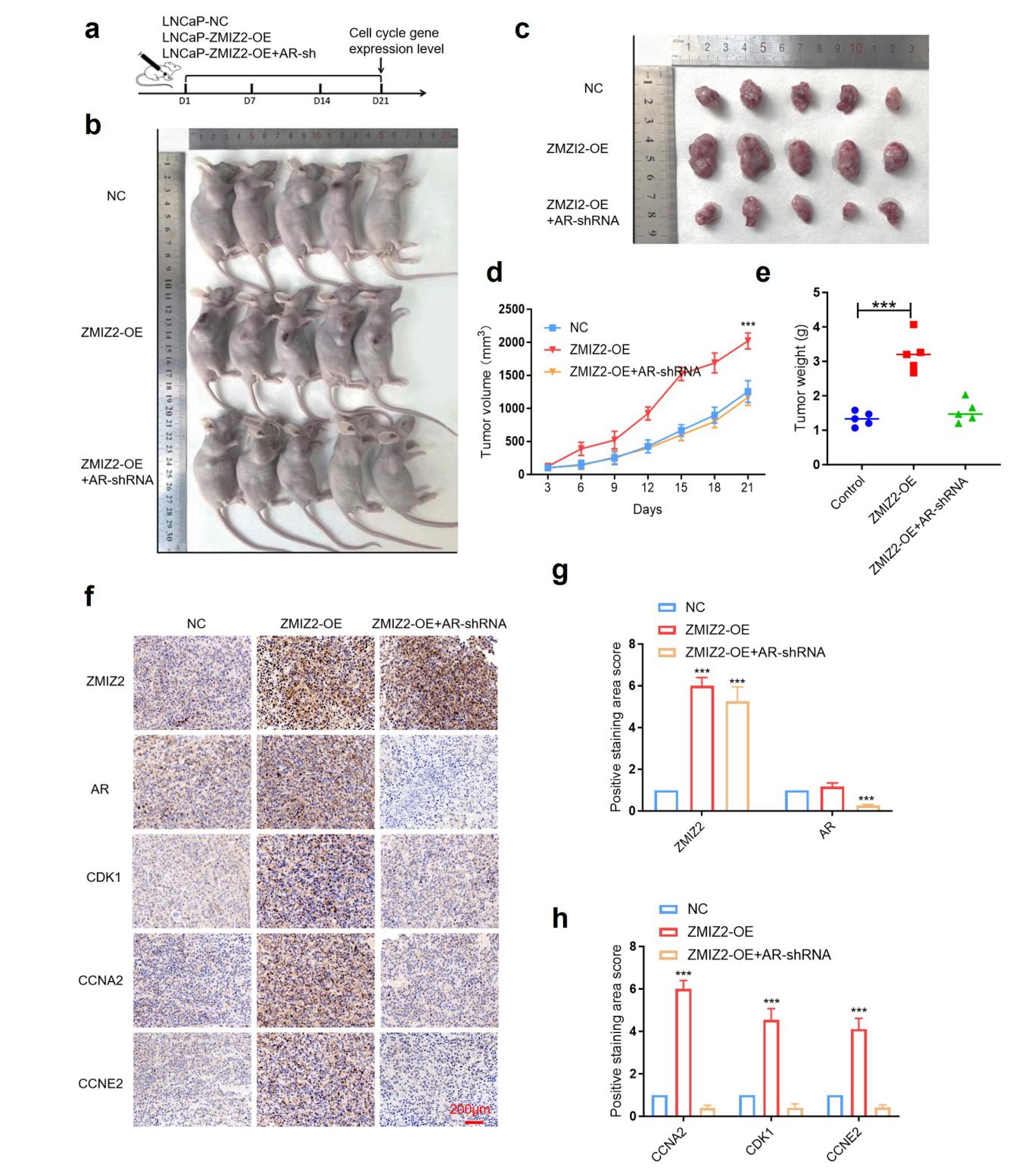


**Figure S2.** LNCaP cells transfected with ZMIZ2 - OE and ZMIZ2 - OE+AR - shRNA were subcutaneously inoculated in nude mice to observe the tumor proliferation rate. (a) LNCaP cells were subcutaneously inoculated in nude mice. Three weeks later, the nude mice were anesthetized with pentobarbital sodium, and the tumors were surgically removed. (b) Photos of nude mice subcutaneously inoculated with LNCaP cells. (c) Tumor images of each group. (d) Volumes of subcutaneous tumors in nude mice of each group recorded at different time points. (e) Tumor masses of mice in each group. (f) Immunohistochemical (IHC) staining of tumor sections for ZMIZ2, AR, and cell cycle-related proteins. (g - h) Densitometric quantification of IHC results. Significant differences are indicated as: **p* < 0.05, ***p* < 0.01, and ****p* < 0.001; ns indicates not significant; n = 5.
