# Supplementary material for: Interaction between ZMIZ2 and AR promotes prostate cancer proliferation in vitro and in vivo
Source: Cancer Biol Ther. 2025 Dec 23;27(1):2604936. doi: 10.1080/15384047.2025.2604936 (PMC12758332; doi:10.1080/15384047.2025.2604936)
Supplement: supplementary material — KCBT_S_2025_0764.R1_Source_Files. [file KCBT_A_2604936_SM6362.zip › 校稿可编辑图片/Supplementary Data/Supplementary Figure 1/Figure Legend.docx]

**Figure S1.** The binding positions of AR and H3K27ac on the promoters of downstream target genes highly overlap. (a - c) The binding sites of AR and the H3K27ac sites on the promoters of CDK1, CCNA2, and CCNE2.
