# Supplementary figures and images for: Interaction between ZMIZ2 and AR promotes prostate cancer proliferation in vitro and in vivo (part 2 of 2)
Source: Cancer Biol Ther. 2025 Dec 23;27(1):2604936. doi: 10.1080/15384047.2025.2604936 (PMC12758332; doi:10.1080/15384047.2025.2604936)

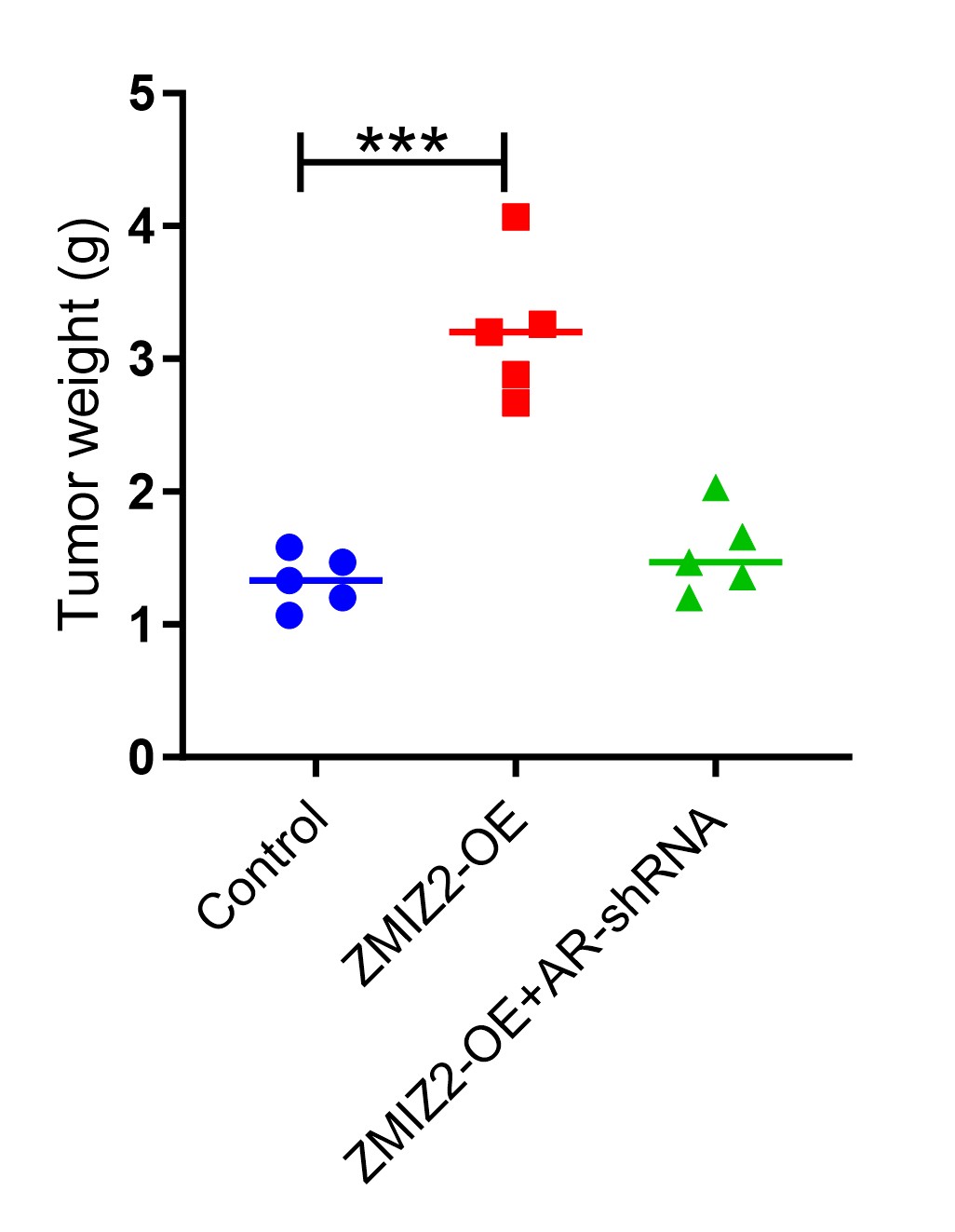

Supplement: supplementary material — KCBT_S_2025_0764.R1_Source_Files. [file KCBT_A_2604936_SM6362.zip › 校稿可编辑图片/Supplementary Data/Supplementary Figure 2/Figure S2e.jpg]

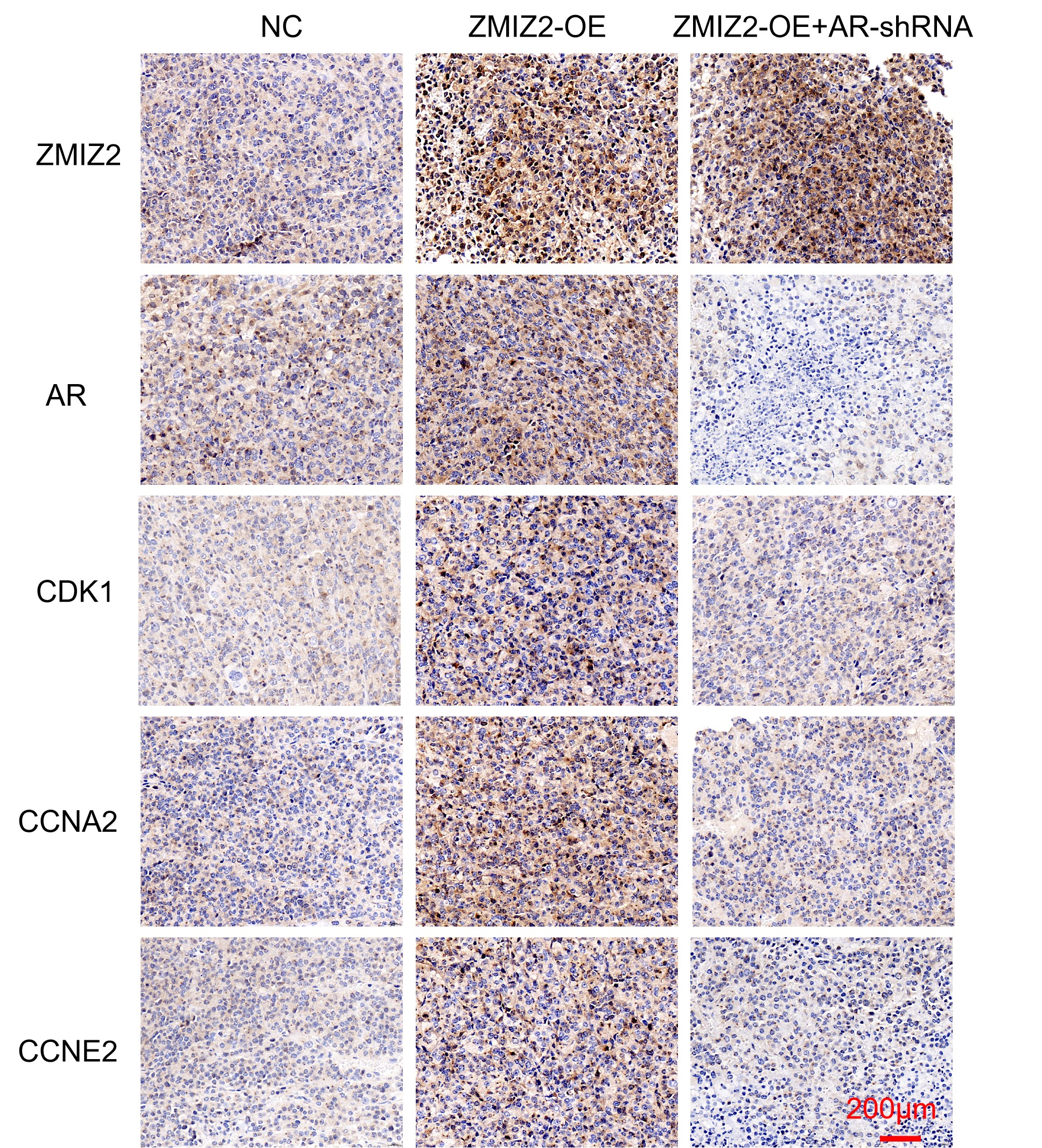

Supplement: supplementary material — KCBT_S_2025_0764.R1_Source_Files. [file KCBT_A_2604936_SM6362.zip › 校稿可编辑图片/Supplementary Data/Supplementary Figure 2/Figure S2f.jpg]

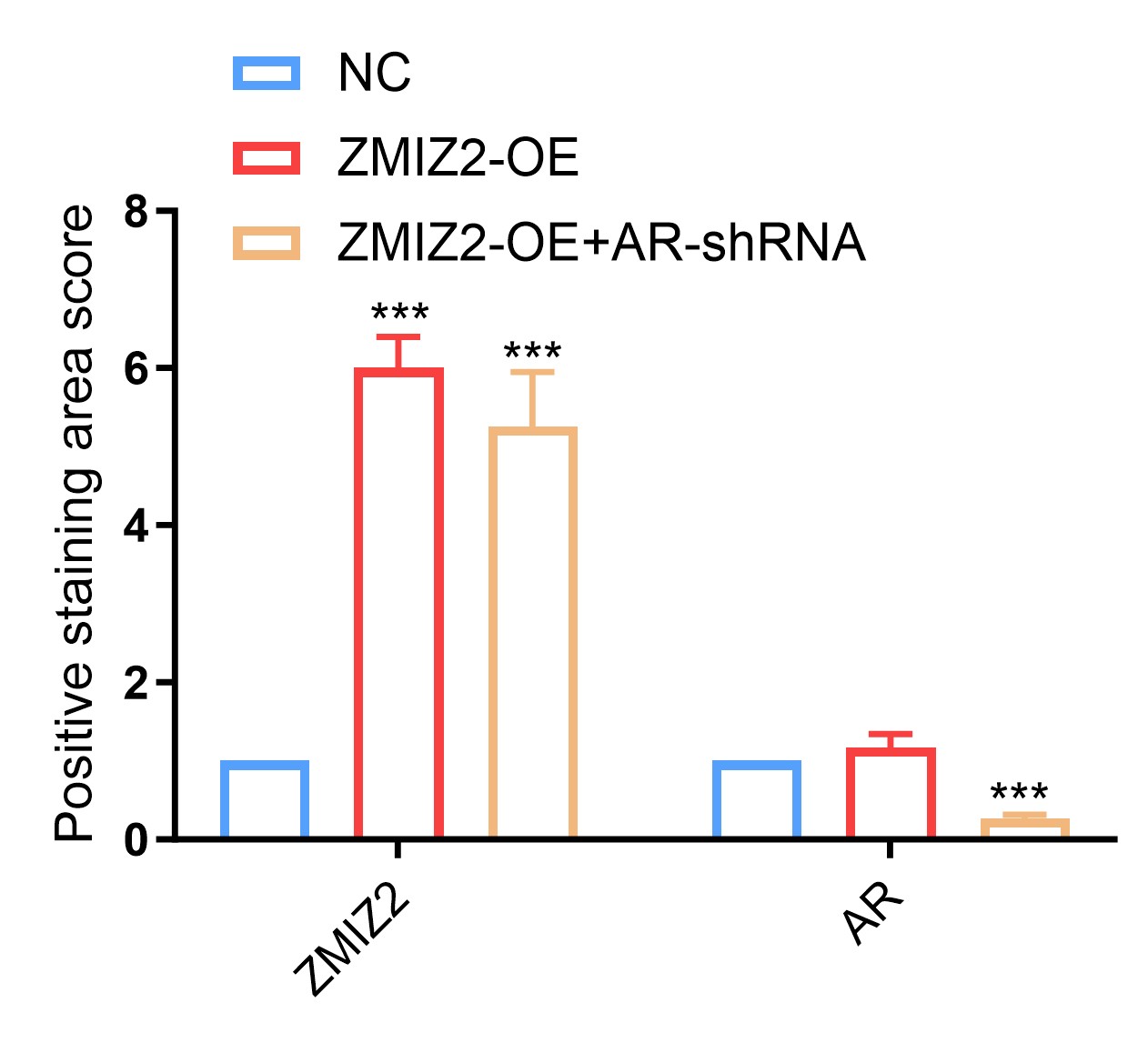

Supplement: supplementary material — KCBT_S_2025_0764.R1_Source_Files. [file KCBT_A_2604936_SM6362.zip › 校稿可编辑图片/Supplementary Data/Supplementary Figure 2/Figure S2g.jpg]

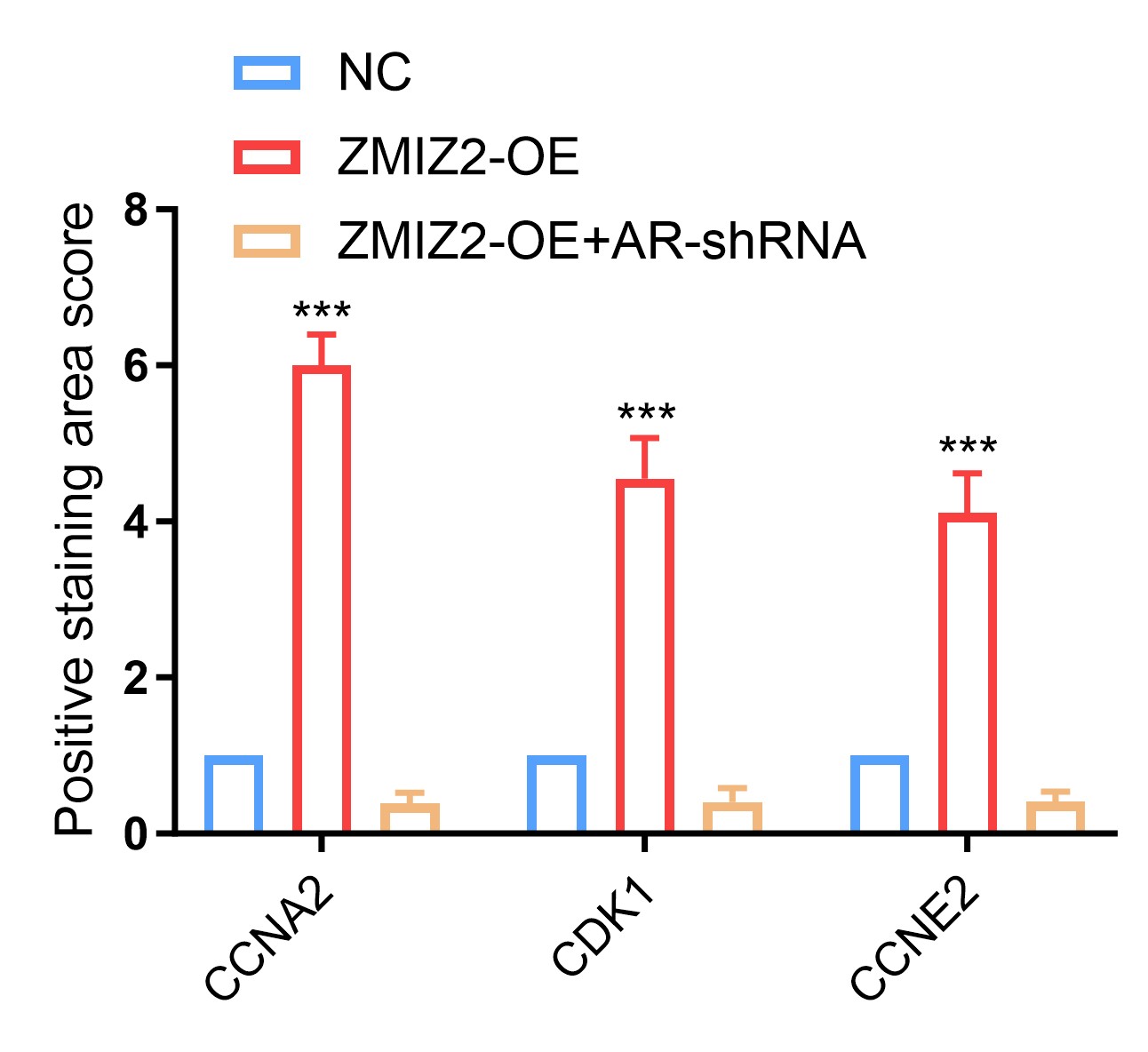

Supplement: supplementary material — KCBT_S_2025_0764.R1_Source_Files. [file KCBT_A_2604936_SM6362.zip › 校稿可编辑图片/Supplementary Data/Supplementary Figure 2/Figure S2h.jpg]
