# Supplementary material for: Interaction between ZMIZ2 and AR promotes prostate cancer proliferation in vitro and in vivo
Source: Cancer Biol Ther. 2025 Dec 23;27(1):2604936. doi: 10.1080/15384047.2025.2604936 (PMC12758332; doi:10.1080/15384047.2025.2604936)
Supplement: supplementary material — Highlights. [file KCBT_A_2604936_SM6359.docx]

Highlights

1. This study identified the functional domain of the interaction between ZMIZ2 and AR protein and elucidated its spatial conformation, laying a structural foundation for revealing the mechanism by which ZMIZ2 synergizes with AR signaling to promote the progression of prostate cancer.
2. This study demonstrated that in prostate cancer cells, ZMIZ2, as a transcriptional co - regulator of AR, recruits acetyltransferases to bind to AR, upregulates the transcriptional activity of downstream target genes, and activates AR signaling, revealing the molecular mechanism by which AR signaling promotes the progression of prostate cancer.
3. This study provides a new theoretical basis and potential molecular targets for prostate cancer treatment strategies targeting the interaction between ZMIZ2 and AR, with significant translational medical value.
